# Ocular Findings Associated With Myelinated Retinal Nerve Fibers

**DOI:** 10.7759/cureus.14552

**Published:** 2021-04-18

**Authors:** Jeslin Kera, Airaj F Fasiuddin

**Affiliations:** 1 Ophthalmology, University of Central Florida College of Medicine, Orlando, USA; 2 Ophthalmology, Nemours Children’s Hospital, Orlando, USA

**Keywords:** mrnf, myelinated retinal nerve fiber layer, optic nerve myelination, amblyopia, strabismus, high myopia, leukocoria, anisometropia

## Abstract

The case involves a five-year-old female patient with a myelinated retinal nerve fiber (MRNF) layer of the right optic disc. Although this is a rare, benign, and often asymptomatic condition, it is sometimes associated with ocular findings which require early detection and treatment. In this case, the patient presented with strabismus, high myopia, and amblyopia. She was found to have myelinated retinal fiber layer lesions of the superotemporal and inferotemporal retina of her right eye. This case report aims to demonstrate the importance of performing a thorough evaluation of MRNF in the pediatric patient as well as to increase awareness of this entity to avoid misdiagnosis.

## Introduction

Myelinated retinal nerve fiber (MRNF) layer is a rare and mostly benign congenital anomaly in which the retinal nerve fibers anterior to the lamina cribrosa have a myelin sheath. Normally, the optic nerve myelination does not extend past the lamina cribrosa and into the retina. Although the direct cause is unknown, MRNF occurs when the myelination extends past this point and is detectable on the fundus examination, obscuring the underlying retinal vessels. This anomaly can be present in up to 1% of the population, and approximately 7% of the affected patients will have bilateral involvement. MRNF typically occurs at birth and involves static lesions. Rarely, it has been reported to be acquired or progressive [1,2].

MRNF was first described in 1856 by a German pathologist, Rudolf Virchow, who described the retina as “white, very thick and wrinkled” [3]. Clinically, the fibers appear as gray-to-white patches with well-defined borders on the anterior surface of the retina. Most cases are diagnosed incidentally in healthy individuals by ophthalmoscopy. Although most children are asymptomatic, few can present with associated ocular findings of high refractive error, strabismus, and amblyopia or failed vision screening. Because myelin blocks light transmission to the underlying retinal cells, there is a high possibility of some vision loss or enlarged blind spot in affected patients [1-3]. MRNF can also present with leukocoria, in which case the differential diagnoses must include the sight-threatening conditions of retinoblastoma, chorioretinitis, coloboma, or cataract.

## Case presentation

**Figure 1:**
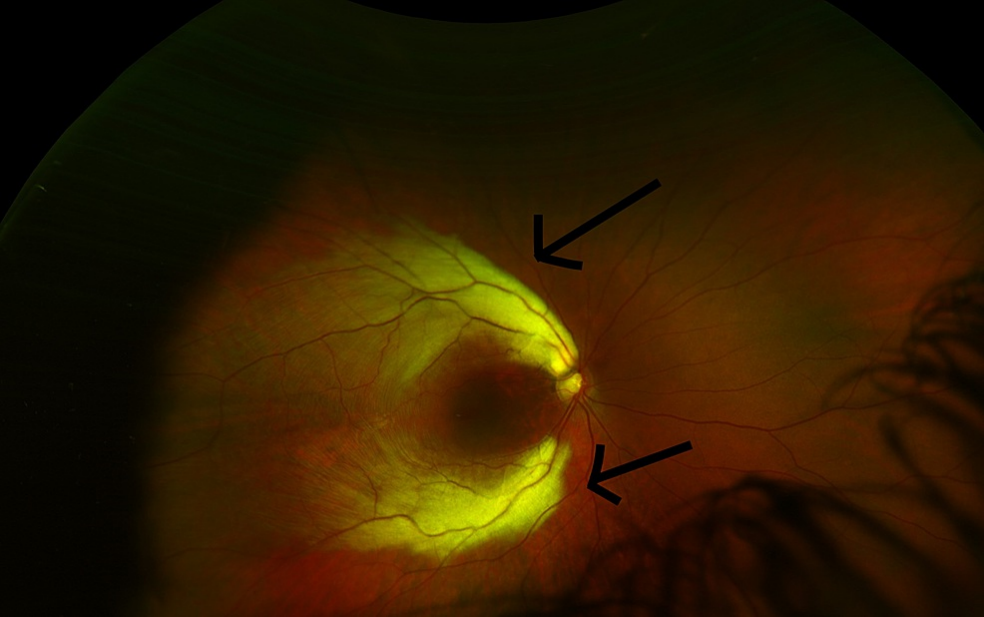
Patient’s right eye. Extensive involvement of both superotemporal and inferotemporal regions of the retina is seen.

**Figure 2:**
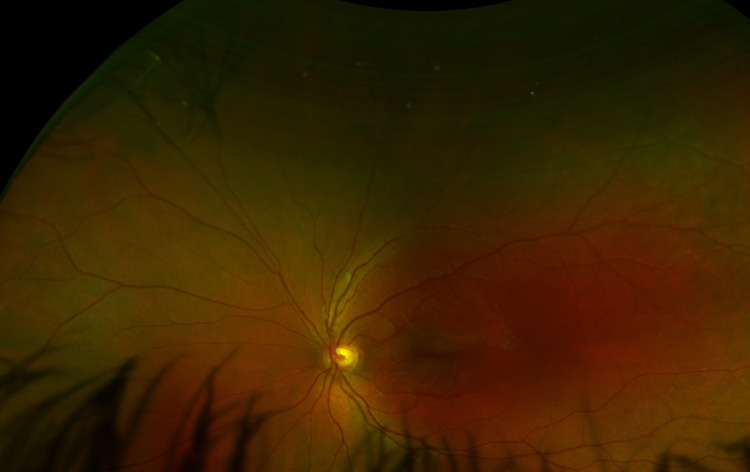
Patient’s left eye. Normal fundus.

This case report presents a five-year-old, otherwise healthy female who was seen in the pediatric ophthalmology clinic for the chief complaint of right eye drifting outward. This occurred especially at distance fixation and was first noticed when the patient was 10 months old. Other significant ocular history included a high refractive error and eyeglasses wear. There was a family history of strabismus in the patient’s maternal uncle. On examination, the patient’s visual acuity was 20/200 in her right eye and 20/40 in her left eye. She was found to have intermittent monocular exotropia of the right eye. She was also found to have a high refractive error in her right eye (-6.50 diopters). The slit lamp examination was normal bilaterally. The dilated fundus examination of the right eye revealed flat, white patches in the retina (superotemporal and inferotemporal regions) in a distribution consistent with a MRNF layer (Figure 1). The left eye, on the other hand, had a normal dilated fundus examination (Figure 2).

The patient was therefore diagnosed with strabismus (intermittent exotropia), high myopia, and amblyopia, as well as MRNF layer of the right eye.

In the patient, the exotropia was a large-sized deviation that was poorly controlled at distance fixation. One year after the initial presentation, she required eye muscle surgery to realign the right eye. The patient was also found to have high myopia in the affected eye. This refractive error caused blurry vision and contributed to her initial visual acuity of 20/200 and the subsequent onset of amblyopia and strabismus. The refractive error was corrected with eyeglasses. Amblyopia treatment was continued with patching of the left eye four hours daily to force the brain to develop vision in the affected right eye.

While patching her left eye, the child was noticed to adopt a head tilt to use her temporal field of vision in the right eye. There was no anomalous head posture noted when not patched and permitted to use both eyes. Because the myelinated nerve fibers cause a blind spot in the affected area, this observed posture was consistent with a nasal visual field defect in the right eye, corresponding to the temporal retinal lesions.

The patient was followed in the pediatric ophthalmology clinic for three years. With the above treatment of strabismus surgery, eyeglasses wear, and patching, the right eye visual acuity improved from 20/200 to 20/50. Her left eye also improved from 20/40 to 20/25. She will continue close follow-up with maintenance patching until at least she is 10 years of age to ensure best potential vision for the affected right eye.

## Discussion

MRNFs are caused by a developmental error involving presence of ectopic oligodendrocyte-like cells in the retina, leading to myelination of retinal ganglion cell fibers. This abnormality occurs when the lamina cribrosa, a protective barrier, is compromised or underdeveloped, allowing oligodendrocytes to penetrate forward into the retina. The impact that MRNF has on the visual functioning is highly variable and may also depend on the extent of myelination patches or if the macula is involved. Macular involvement is less common and can be symptomatic for severe photophobia. Optic nerve abnormalities, such as dysplastic or hypoplastic optic nerve, can be coexistent organic pathologies with MRNF and additional causes of further visual impairment [4].

As seen in this patient’s case, MRNF can be associated with multiple ocular findings, and it is important to recognize and treat each accordingly. Most children with MRNF have good visual potential in the affected eye as the macula is generally spared due to its high concentration of ganglion cells and low density of nerve fibers. However, pediatric population can develop amblyopia due to the associated conditions of strabismus, anisometropia, or high myopia. Amblyopia develops when the brain fails to process input from one eye due to refractive error, strabismus, or structural anomaly, and over time brain favors the other eye. This results in varying degrees of vision loss in the affected eye. In cases of amblyopia caused by refractive error or strabismus, the eye may otherwise be structurally normal. Visual development is typically complete by age 10, so early detection and treatment of amblyopia is essential. Refractive amblyopia can be treated with continuous wearing of proper eyeglasses and patching of the healthy eye to force the patient to use the amblyopic eye [5-7]. Strabismic amblyopia is also managed with eye patching and with realignment of the eye, either with eyeglasses and/or with eye muscle surgery. Patient presented in this case required strabismus surgery as part of her amblyopia treatment due to significant exotropia, which eventually helped her vision further improve. She presented with 20/200 vision in the affected eye which improved to 20/50 with the proper treatment. This case demonstrates the impact of prompt diagnosis and treatment to optimize visual potential in childhood.

Although MRNF is typically benign, it is sometimes mistaken for other conditions that are potentially more serious. When presenting in young children as leukocoria detected by a pediatrician, MRNF can be mistaken for retinoblastoma, chorioretinitis, coloboma, or cataract [8]. Prompt referral to an ophthalmologist for a dilated fundus examination is therefore indicated. MRNF is characterized by non-elevated whitening of the affected retina, following the pattern of nerve fiber distribution in the retina. The affected area can be hyperreflective on optical coherence tomography (OCT). Mimicking conditions include retinal vascular occlusion and cotton wool spots, which also show hyperreflectivity on OCT but can be distinguished from MRNF using ophthalmoscopy or fluorescein angiography. Serial fundus examinations help differentiate MRNF from other transient or fluctuating entities, including neurologic, retinal, neoplastic, and inflammatory diseases. These include pituitary adenoma, acute optic neuropathy, optic neuritis, primary open-angle glaucoma, central retinal artery occlusion, branch retinal artery occlusion, diabetic retinopathy, or neoplastic infiltrate [9,10]. Systemic syndromes associated with MRNF include neurofibromatosis type I, Gorlin syndrome, Turner syndrome, trisomy 21, epilepsy, and craniosynostosis syndromes. In some of these syndromes, vascular anomalies such as neovascularization, bleeding, or thrombus development have been found in the region of the myelinated nerve fibers, creating another potential etiology for vision loss that may require treatment [3].

## Conclusions

Although MRNF is a benign condition by itself, it may be associated with ocular findings that warrant prompt and appropriate evaluation and treatment. In childhood, this is especially time-sensitive due to the finite period of visual development and the potential for response to amblyopia treatment. Commonly associated findings that cause amblyopia include high refractive error, strabismus, and anisometropia. MRNF may also present as leukocoria, which requires an immediate ophthalmologic evaluation to identify any sight-threatening ocular conditions. MRNF typically has a good prognosis if associated ocular findings are promptly addressed, as witnessed in this presented case.
